# Clinical neuropathology practice news 2-2012: BRAF V600E testing 

**DOI:** 10.5414/NP300492

**Published:** 2012-02-29

**Authors:** David Capper, Anna-Sophie Berghoff, Andreas von Deimling, Matthias Preusser

**Affiliations:** 1 Department of Neuropathology, Institute of Pathology, Ruprecht-Karls-University Heidelberg,; 2 Clinical Cooperation Unit Neuropathology, German Cancer Research Center (DKFZ), Heidelberg, Germany; 3 Institute of Neurology; 4 Department of Medicine I and Comprehensive Cancer Center CNS Unit, Medical University of Vienna, Austria

**Keywords:** BRAF, BRAF V600 mutation, immunohistochemistry, melanoma, brain metastases

## Abstract

Activating mutations of the serine threonine kinase v-RAF murine sarcoma viral oncogene homologue B1 (BRAF), most commonly of the V600E type, are found in a wide range of human neoplasms including primary and secondary brain tumors. Therapeutic BRAF inhibitors have shown clinically meaningful activity, particularly in metastatic BRAF V600E mutated melanoma including patients with brain metastases. Therefore, in current neuropathological practice BRAF testing is of clinical importance in tissue samples of melanoma brain metastases in order to identify cases amenable to therapy with BRAF inhibitors. BRAF mutation testing may also add additional information for differential diagnosis of primary brain tumors in selected situations, e.g., for differentiation of anaplastic pleomorphic xanthoastrocytoma (BRAF V600E mutation in 65%) from glioblastoma (BRAF V600E mutation in < 5%). The BRAF mutation status can be tested with DNA-based methods and immunohistochemistry using a V600E mutation-specific antibody. In summary, at this point BRAF V600E testing is clinically indicated in relatively few cases of the daily clinical neuropathology practice, but has important predictive implications for patients with melanoma brain metastases. Depending on the results of additional clinical studies, determination of BRAF mutation status may become clinically relevant also for primary brain tumors such as glioblastoma in the future.

## Background and rationale for BRAF V600E mutation testing 

Activating mutations of the serine threonine kinase v-RAF murine sarcoma viral oncogene homologue B1 (BRAF) are found in a wide range of human cancers and represent the most frequent unique genetic alteration among melanoma (50 – 60%), papillary thyroid carcinoma (40 – 70%) and hairy cell leukemia (> 90%) [1, 2]. Among primary brain tumors, activating BRAF point mutations are found frequently in pleomorphic xanthoastrocytoma (60 – 70%) and ganglioglioma (~ 20%) and less frequently in pilocytic astrocytomas [3]. For primary glioblastomas this alteration is altogether rare (< 5%) but is more frequent in the subset of pediatric glioblastomas (~ 10%). More than 95% of BRAF mutations are of the V600E type, which leads to the substitution of valine by glutamic acid in the activating segment of the kinase domain of BRAF. The mutated BRAF protein is constitutively activated and enhances the proliferative potential through activation of the mitogen-activated protein kinase (MAPK) pathway [4]. BRAF mutations are distinct from the KIAA1549:BRAF fusion and other types of BRAF fusions, alterations found frequently especially in cerebellar pilocytic astrocytomas [5]. 

Testing for the presence of BRAF V600E mutation may be relevant in clinical neuropathology practice for the following reason: BRAF mutations affect some brain tumors (both primary and secondary) [3, 6] and small molecule drugs specifically inhibiting the mutated BRAF protein have been developed. These small molecules have shown clinically meaningful activity in metastatic BRAF V600E mutated melanoma including patients with brain metastases [7, 8, 9]. 

## Whom to test? 

In current neuropathological practice, BRAF testing can be of clinical value in tissue samples of melanoma brain metastases from patients with unknown mutation status in order to identify cases amenable to therapy with BRAF inhibitors. The BRAF status has been shown not to vary between different tumor manifestations of the same patient [6]. Thus, in patients with previously tested BRAF status of the primary tumor or extracranial metastases, no additional analysis of the brain metastasis is required. In selected situations, BRAF mutation testing may also add additional information for the differential diagnosis of primary brain tumors, e.g., for differentiation of anaplastic pleomorphic xanthoastrocytoma from glioblastoma or giant cell glioblastoma [3]. 

## How to test? 

The BRAF mutation status can be analyzed using DNA-based methods such as the US Food and Drug Administration (FDA) approved Cobas 4800 BRAF V600 test (Roche, Pleasanton, CA, USA), which is commercially available. Recently, a monoclonal antibody (antibody VE1) which reliably detects BRAF V600E mutated protein in formalin-fixed and paraffin-embedded tumor tissue samples has been generated [10]. VE1 antibody has shown high specificity and sensitivity in several tumor types [6, 11]. The commercialization of the VE1 antibody is ongoing. A sequential algorithm using initial immunohistochemical testing and DNA-based validation in unclear cases may combine maximum practicability and optimize diagnostic accuracy. 

## Conclusion 

In summary, at this point BRAF V600E testing is clinically indicated in relatively few cases of the daily clinical neuropathology practice, but has important therapeutic implications in selected patients. It seems advisable that implementation of the test should be a joint decision of (neuro-)pathologists and (neuro-)oncologists at a given center. 

## Conflict of interest 

Andreas von Deimling and David Capper declare shared inventorship of BRAF antibody clone VE1. A patent for diagnostic application of VE1 has been applied for. All terms are being managed by the German Cancer Research Center in accordance with its conflict of interest policies. Dr. Preusser has recieved lecture honoraria and travel and research support by Roche Austria. 
